# Clinical Benefits of *Aloe vera* Gel in 0.3% Hyaluronate Eyedrops in Glaucoma Therapy-Related Ocular Surface Disease

**DOI:** 10.3390/biomedicines14010186

**Published:** 2026-01-15

**Authors:** Luca Agnifili, Davide Celani, Alessandro Sferra, Maria Ludovica Ruggeri, Rodolfo Mastropasqua, Michele Figus, Matteo Sacchi

**Affiliations:** 1 Ophthalmology Clinic, Department of Medicine and Ageing Science, “G. d’Annunzio” University Chieti-Pescara, 66100 Chieti, Italy; 2 Alma Mater Studiorum, University of Bologna, 40126 Bologna, Italy; 3 Department of Neuroscience, Imaging and Clinical Science, “G. d’Annunzio” University Chieti-Pescara, 66100 Chieti, Italy; 4 Eye Clinic, University Hospital of Chieti-Pescara, 66100 Chieti, Italy; 5 Ophthalmology Unit, Department of Surgery, Medicine, Molecular and Emergency, University of Pisa, 56127 Pisa, Italy; 6 Department of Medicine, Surgery and Pharmacy, University of Sassari, 07100 Sassari, Italy

**Keywords:** glaucoma, ocular surface, GTOSD, inflammation

## Abstract

**Background**: *Aloe vera* gel in 0.3% hyaluronate (AV/HA) could mitigate glaucoma therapy-related ocular surface disease (GTOSD). **Methods**: Thirty-nine patients diagnosed with GTOSD and receiving AV/HA or HA underwent ocular surface disease index (OSDI), Symptom Assessment iN Dry Eye (SANDE), National Eye Institute Visual Function Questionnaire (NEI VFQ)-25 questionnaires, and tear matrix metalloproteinase-9 (MMP-9), break-up time (BUT), corneal fluorescein staining (CFS), Schirmer test I (STI), and bulbar conjunctival hyperemia (BCH) determination. **Results**: After one month, *AV/HA* increased BUT (5 (7–4.5) to 7 (8–5.5)) and STI (12 (19.5–8) to 13.5 (20–10)), while it decreased BCH (2.2 (2.3–1.3) to 2.1 (2.2–1.2)) and CFS (3 (4–2) to 2 (3.0–1.5)) (*p* < 0.001). SANDE and OSDI scores were reduced from 36.18 (38.5–20.5) to 22.91 (31.5–17.21), and 29.5 (32.5–19.5) to 20 (26.5–18) (*p* < 0.001). *HA* reduced BCH from 2.75 (3.20–2.15) to 2.25 (2.30–1.90) (*p* = 0.014) and CFS from 3.5 (5–2.75) to 2.5 (4–2) (*p* = 0.014), while it increased BUT (*p* = 0.036). The SANDE score decreased from 28.95 (47.6–20.9) to 26.86 (36.41–19.90) (*p* = 0.009), whereas the OSDI decreased from 40 (49–19.5) to 29 (42–19.75) (*p* = 0.005). Any significant change in NEI VFQ-25 was collected. A trend for an MMP-9 immunoassay positivity reduction was observed in AV/HA (0.073). **Conclusions**: These findings invite considering lubricants enriched with natural anti-inflammatory agents, such as *Aloe vera*, as a potential adjunctive option to improve the ocular surface in glaucoma.

## 1. Introduction

Topical intraocular pressure (IOP) medications produce undesirable effects on the ocular surface in a high proportion of patients with glaucoma [1]. Epithelial damage, loss of glandular components, tear film instability, and chronic inflammation, are the main features of glaucoma therapy-related glaucoma ocular surface disease (GTOSD), which is an iatrogenic form of dry eye [2,3,4].

The recognition of GTOSD is of crucial importance, as the disease may worsen the adherence to therapy, disease control, and quality of life (QoL), and may negatively affect filtration surgery outcomes [3,4]. Because of this, GTOSD deserves dedicated management.

Eyelid margin status improvement and the mitigation of dry eye and inflammation represent key steps, as they are some of the pivotal features of GTOSD [2,3,4]. Thus, lubricants and anti-inflammatory agents are frequently prescribed as supporting therapies during the course of the disease.

Among lubricants, high-molecular-weight hyaluronic acid (HMW-HA) is one of the most performant, since, in addition to its mucoadhesive, mucomimetic, and water-retaining properties, it increases goblet cell density, and MUC5AC secretion, and tends to contain ocular surface inflammation [5,6]. Considering inflammation, topical steroids are highly effective, but most of them are unsuitable for glaucoma. Therefore, alternative strategies have to be considered to face this aspect of the GTOSD.

*Aloe vera* is a widely recognized herbal medicine, used as an effective natural remedy for many clinical conditions [7,8,9,10]. The presence of several active compounds within the extracts provides *Aloe vera* with essential biological properties, such as anti-microbial effects, wound healing, antioxidant effects, wetting capability, immune-modulation, and anti-inflammatory effects [7,8,9,10,11,12].

The anti-inflammatory effects of *Aloe vera* extracts are especially obtained through the reduction in cytokine and matrix metalloproteinases (MMPs) production [11,13,14]. As validation of these properties, a recent prospective, randomized, double-masked, and placebo-controlled study conducted in patients with dry eye showed that lubricants containing *Aloe vera* reduced the MMP-9 and IL-6 concentrations in tears, and improved the break-up time (BUT) values and the dry eye questionnaire (DEQ) score [11]. Although glaucoma therapy-related ocular surface disease represents a distinct, iatrogenic condition compared with primary dry eye disease, they share a persistent inflammatory process, thus providing the rationale for also exploring *Aloe vera*-based adjunctive treatments in patients with GTOSD. Taken together, these results suggest a potential application of eyedrops containing *Aloe vera* extracts in mitigating dry eye and inflammation in different forms of OSD. On this basis, we hypothesized that a hyaluronate-based lubricant enriched with *Aloe vera* could provide clinical benefits in GTOSD by modulating ocular surface inflammation beyond lubrication alone. Therefore, we conducted the present study to evaluate the clinical, quality-of-life-related, and anti-inflammatory effects of a preservative-free (PF) fixed combination of *Aloe vera* gel in 0.3% hyaluronate (*Aloe vera*/0.3% HA), used as a short-term adjunctive therapy in patients with medically controlled glaucoma and GTOSD.

Given the real-world, multifactorial, and iatrogenic nature of GTOSD, and the absence of previous studies evaluating *Aloe vera*-containing formulations in this specific setting, an observational sub-analysis within a dedicated glaucoma ocular surface registry (OSGLA) represents an appropriate approach for exploring the clinical relevance of this strategy in a medically controlled glaucoma population.

## 2. Materials and Methods

### 2.1. OSGLA Registry

#### 2.1.1. Study Design (OSGLA Registry)

The OSGLA Registry is an ongoing, prospective, multicenter, observational, and non-interventional study designed to collect standardized, longitudinal data on ocular surface alterations in glaucomatous patients controlled with medical therapy. The registry was approved by the Institutional Review Board of the Department of Medicine and Ageing Science, University “G. d’Annunzio” of Chieti-Pescara (approval no. 2024.0121). The OSGLA Registry was designed to monitor the overall systemic and ocular characteristics of patients with GTOSD, with particular attention to clinical, molecular, instrumental, and clinical ocular surface parameters, quality-of- life-related questionnaires, and treatment regimens, regularly collecting follow-up data over time. Centers involved in the registry include patients consecutively, according to a shared visit schedule. Because of its observational, non-interventional design, the OSGLA Registry does not provide recommendations regarding therapy; treatment regimens are modified based on clinical judgment and are regularly recorded in the OSGLA Registry datasheet. The registry complies with the Declaration of Helsinki, and all participants provide written informed consent before enrollment.

#### 2.1.2. Registry Outcomes (OSGLA Registry)

The primary outcomes of the OSGLA Registry are measurements of longitudinal changes in the ocular surface disease severity of GTOSD, assessed by the ocular surface disease index (OSDI) score, tear film break-up time (BUT), Schirmer’s test I (STI), corneal fluoresceine staining (CFS) using the Oxford scheme, bulbar conjunctiva hyperemia (BCH) using McMonnies/Chapman-Davies scale, and tear matrix metalloproteinase (MMP)-9 immunoassay positivity. Secondary outcomes include Symptom Assessment iN Dry Eye (SANDE) and National Eye Institute Visual Function Questionnaire (NEI VFQ)-25 scores, IOP, visual field and optic nerve parameters, adherence to therapy information, treatment switches, and surgical events.

For the OSGLA Registry, inclusion criteria were as follows: aged 18 years or more, diagnosis of POAG (visual field test (Humphrey field analyzer II 750 (Carl Zeiss Meditec Inc., Dublin, CA, USA) (24 − 2 test, full-threshold)) showing at least three contiguous points on the total deviation probability plot at a less-than-2% level, Glaucoma Hemifield Test “outside normal limits” and signs of glaucomatous optic disc consistent with the perimetry), IOP controlled with topical medical therapy (mean of three measurements taken at 9 AM; Goldmann applanation tonometry), and visual field stability in the last 12 months. The diagnosis of GTOSD required an OSDI score > 13 and alterations in clinical ocular surface tests (BUT < 10 s, positive CFS, or BCH ≥ grade 1) [15,16,17].

*Exclusion criteria* were as follows: previous ocular surgery (including laser refractive surgery), cataract surgery performed less than six months before enrollment, trauma, concomitant ocular diseases other than glaucoma and GTOSD, any concomitant topical and/or systemic therapy potentially modifying the ocular surface or the tear film including topical lubricant and anti-inflammatory agents, Sjogren’s syndrome, thyroid disease or any other auto-immune disease, allergy, diabetes mellitus, use of oral contraceptives, non-adherence to medical therapy for glaucoma, pregnancy, breastfeeding, contact lens wearing, instillation of lubricants in the last month.

#### 2.1.3. Protocol (OSGLA Registry Protocol)

As per protocol, patients enter the Registry undergoing a comprehensive assessment including the following: (i) questionnaire score determinations: OSDI, SANDE, and NEI VFQ-25; (ii) ocular surface clinical test execution: BUT, CFS, BCH, and STI); (iii) ocular surface molecular biomarker determination: tear MMP-9 levels; (iv) clinical-related parameter collection, such as modifications of IOP and IOP-lowering therapy, introduction of concomitant topical or systemic therapies, disease progression, adverse events, adherence to therapy, and surgical outcomes.

Visits are scheduled at baseline, month 1 (±7 days), after major therapeutic modifications (including the introduction of concomitant therapies such as lubricants, anti-inflammatory agents, or antiglaucoma regimen variations), and every 4 months (±30 days) thereafter, with possible variations according to the clinical judgment.

### 2.2. Current Study: Lubricant Cohort Sub-Analysis

This prospective sub-analysis included OSGLA Registry participants who initiated lubricants as adjunctive therapy to manage GTOSD. In detail, patients treated with PF *Aloe vera* gel/0.3% HA eyedrops (Micuro Intensive Eyedrop-Gel, 4Health Pharma, Bologna, Italy) or with PF 0.3% HA alone (ESO DROP, Esoform, Rovigo, Italy) who completed both the baseline and 1-month follow-up visits according to the registry schedule were considered. The dose regimen of each treatment was one eyedrop per eye, three times a day.

#### 2.2.1. Outcomes

Primary outcomes were the within-group changes from baseline to month 1 of the BUT, STI, CFS, BCH, and questionnaire scores; the secondary outcome was the tear MMP-9 immunoassay positivity changes at follow-up. Correlations between MMP-9 immunoassay positivity and ocular surface clinical tests and questionnaire scores at follow-up were also explored. All assessments followed the standardized OSGLA Registry procedures and were performed by the same investigator at each center.

#### 2.2.2. Statistical Analysis

Statistical analyses were performed using the R software environment for statistical computing and graphics (version 4.4.3 (28 February 2025 ucrt); http://www.r-project.org/).

Normality of data distribution was assessed using the Kolmogorov–Smirnov test, the Shapiro–Wilk test, the Jarque–Bera test, and by visual inspection of Q-Q plots. Given the non-normal distribution of most variables, non-parametric methods were adopted. Descriptive statistics were then reported as median and interquartile range (IQR) [first quartiles; third quartiles].

Specifically, the Wilcoxon signed-rank test was used to compare pre- and post-treatment values in *Aloe vera* gel/0.3% HA and 0.3% HA.

To quantify the magnitude of pre–post treatment changes, effect sizes (r) were calculated for all ocular surface parameters and questionnaire scores. According to Cohen’s criteria, effect sizes were classified as small (r ≈ 0.1), moderate (r ≈ 0.3), and large (r ≥ 0.5).

Boxplots were generated to illustrate the statistically significant differences. For the binary variable MMP-9 levels (positive vs. negative), comparisons were performed using the McNemar test to assess changes between baseline and follow-up within each treatment group. Correlation analyses between MMP-9 immunoassays were conducted using Spearman’s rank correlation coefficient, due to the non-normal distribution of the variables, as confirmed by multiple normality tests.

The empirical power (1 − β) was estimated at 0.922, indicating a high probability of correctly detecting a true effect; the Type-II error rate (β) was correspondingly low at 0.078; and the Type-I error rate (α) was consistent with conventional thresholds at 0.048. All statistical tests were two-sided, and a *p*-value ≤ 0.05 was considered statistically significant.

## 3. Results

Thirty-nine patients from the Glaucoma Centre of the University of Chieti-Pescara, Chieti, Italy, were included in this sub-analysis. Of them, 12 were treated with PF HA 0.3% and 27 with PF *Aloe vera*/0.3% HA. Baseline demographic and clinical features of patients are reported in Table 1. Overall, follow-up outcomes revealed a statistically significant improvement for most of the ocular surface parameters and questionnaire scores in both treatment regimens, with numerically larger within-group changes observed in patients receiving PF *Aloe vera*/0.3% gel HA (Table 2)

### 3.1. PF HA 0.3% Treatment Regimen

**Figure 1:**
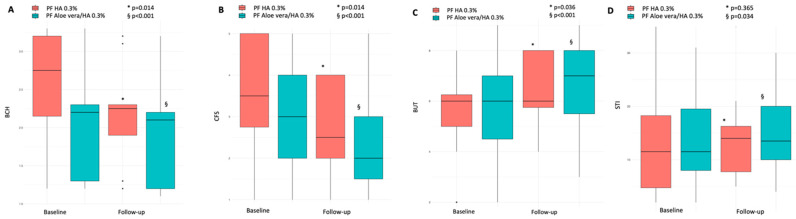
Baseline and one-month BCH (**A**), CFS (**B**), BUT (**C**), and STI (**D**) values in PF *Aloe vera*/0.3% HA and PF 0.3% HA treatments. Variables improved in both treatments, with numerically greater improvements observed in patients treated with PF *Aloe vera*/0.3% HA. BCH: bulbar conjunctiva hyperemia; CFS: corneal fluoresceine staining; BUT: break-up time; STI: Schirmer test I; PF: preservative-free; HA: hyaluronic acid.

**Figure 2:**
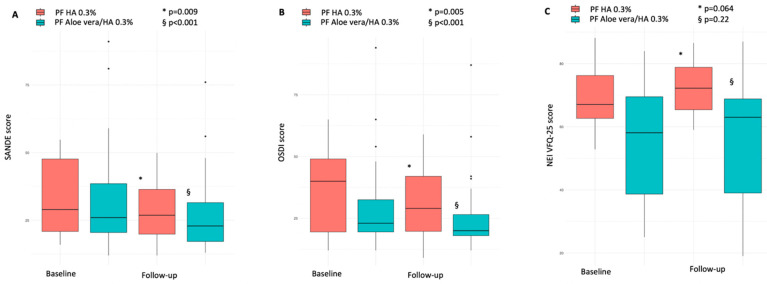
Baseline and one-month SANDE (**A**), OSDI (**B**), and NEI VFQ-25 (**C**) scores in PF *Aloe vera*/0.3% HA and PF 0.3% HA treatments. SANDE and OSDI scores improved in both treatments, with numerically greater improvements observed in patients treated with PF *Aloe vera*/0.3% HA. SANDE: Symptoms Assessment iN Dry Eye; OSDI: ocular surface disease index; NEI VFQ-25: National Eye Institute Visual Function Questionnaire; PF: preservative-free; HA: hyaluronic acid.

After one month of therapy, PF HA 0.3% eyedrops significantly improved both BCH and CFS, with median values decreasing by almost 20%, from 2.75 (3.20–2.15) to 2.25 (2.30–1.90) (*p* = 0.014), and 30%, from 3.5 (5–2.75) to 2.5 (4–2) (*p* = 0.014), respectively. Though the median value remained numerically unchanged, the BUT also showed a significant improvement (*p* = 0.036). Conversely, no significant changes were observed for the STI (*p* = 0.365) (Figure 1A–D). When considering questionnaire scores, while those for SANDE decreased from 28.95 (47.6–20.9) to 26.86 (36.41–19.90) (*p* = 0.009), and for OSDI declined from 40 (49–19.5) to 29 (42–19.75) (*p* = 0.005), scores for NEI-VFQ25 did not significantly change (*p* = 0.064) (Figure 2A–C). A reduction in the positivity of the MMP-9 immunoassay was observed (from 8 to 6), but this change was not statistically significant (*p* = 0.479).

In the PF HA 0.3% group, large effect sizes were observed for BCH (r = 0.71), CFS (r = 0.70), SANDE (r = 0.75), BUT (r = 0.60), OSDI (r = 0.81), and MMP-9 immunoassay (r = 0.50). The effect size for NEI-VFQ25 was moderate (r = 0.53), while STI showed a small-to-moderate effect (r = 0.26).

### 3.2. PF Aloe vera/0.3% HA Treatment Regimen

In this treatment regimen, follow-up improvements were, for the most part, broader. BCH values slightly decreased, from 2.2 (2.3–1.3) to 2.1 (2.2–1.2) (*p* < 0.001). BUT increased by almost 30%, from 5 (7–4.5) to 7 (8–5.5) seconds (*p* < 0.001), STI increased by 12%, from 12 (19.5–8) to 13.5 (20–10) mm (*p* = 0.034), and CFS scores decreased by a third, from 3 (4–2) to 2 (3.0–1.5) (*p* < 0.001) (Figure 1A–D). When considering questionnaires, both the SANDE and OSDI scores significantly improved, with values decreasing by more than a third, from 36.18 (38.5–20.5) to 22.91 (31.5–17.21) (*p* < 0.001) and 29.5 (32.5–19.5) to 20 (26.5–18) (*p* < 0.001), respectively. Similar to the PF HA 0.3% treatment, NEI-VFQ25 scores increased, but the change was not statistically significant (*p* = 0.22) (Figure 2A–C). Finally, though a reduction in the positivity of the MMP-9 immunoassay was observed (from 16 to 11), and the trend was toward statistical significance, this was not reached (*p* = 0.0736). None of the patients, in either treatment strategy, reported any topical or systemic adverse effects during treatments.

In the PF *Aloe vera*/0.3% HA group, large effect sizes were found for BCH (r = 0.72), CFS (r = 0.69), SANDE (r = 0.74), BUT (r = 0.69), OSDI (r = 0.72), and MMP-9 immunoassay (r = 0.80). STI showed a moderate effect (r = 0.41), whereas NEI-VFQ25 showed a small effect (r = 0.23).

Between-group comparisons of pre–post treatment differences revealed no statistically significant differences for any of the evaluated parameters, including BCH (*p* = 0.41), STI (*p* = 0.68), CFS (*p* = 0.44), SANDE (*p* = 0.81), BUT (*p* = 0.67), NEI-VFQ25 (*p* = 0.82), and OSDI (*p* = 0.47). Although numerically larger improvements were observed in the PF *Aloe vera*/0.3% HA group for several outcomes, these differences did not reach statistical significance, likely due to the relatively small sample size and variability within the groups. These findings suggest that, while both treatments provide clinically meaningful benefits, the comparative efficacy between the two cannot be definitively established in this non-randomized cohort.

Although intra-group analyses demonstrated large effect sizes for most ocular surface parameters and symptom scores in both treatment regimens, the absence of statistically significant between-group differences suggests that the two treatments provided similar overall clinical benefits. Reporting both effect sizes and between-group *p*-values allows a more comprehensive assessment of treatment effects and supports a balanced interpretation of comparative efficacy.

The present study did not include an a priori sample size calculation, as patient allocation to treatment groups was based on routine clinical practice rather than randomized assignment. Post hoc power analyses were therefore conducted using the observed effect sizes (Cohen’s d) for each outcome measure, assuming a significance level of α = 0.05 and a target power of 80%. Based on these estimates, the sample size required to detect an effect of the observed magnitude ranged from approximately 10 patients for BUT to 23 patients for SANDE, while a substantially larger sample (approximately 97 patients) would have been required for NEI-VFQ25.

Accordingly, the statistical power differed across outcome measures, with NEI-VFQ25 being associated with a higher risk of type-II error compared with other parameters.

Patients were assigned to the PF *Aloe vera*/0.3% HA or PF HA 0.3% treatment groups according to clinical practice rather than by randomization. This non-randomized design may introduce selection bias and limits causal inference, as baseline differences or unmeasured confounding factors, including age, disease severity, or patient and physician preferences, cannot be fully excluded.

In this context, the absence of statistically significant differences between treatment groups should be interpreted cautiously. Randomized controlled trials with prospectively calculated sample sizes are required to more definitively assess the comparative efficacy of PF *Aloe vera*/0.3% HA and PF HA 0.3%.

### 3.3. Correlation Analysis

No statistically significant correlations were observed between post-treatment ocular surface parameters and questionnaire scores and the MMP-9 immunoassay positivity, neither for PF *Aloe vera*/0.3% nor for PF HA 0.3.% treatment.

## 4. Discussion

This study was part of the OSGLA Registry, which was established to collect longitudinal clinical, instrumental, and patient-reported data on the ocular surface alterations in glaucoma patients controlled with medical therapy. Here, we evaluated the short-term effects of two formulations of lubricants containing PF 0.3% hyaluronate, one of them enriched with *Aloe vera*, as adjunctive treatment for GTOSD.

Overall, we found that one month of treatment with *Aloe vera* gel in a 0.3% HA vehicle improved symptoms and signs of GTOSD.

Nevertheless, although patients receiving *Aloe vera*/0.3% HA showed broader improvements across clinical parameters and patient-reported outcomes over HA, these findings should be interpreted with particular caution. In fact, given the observational nature of the study, the absence of randomization, and the lack of formal between-group statistical comparisons, it is not possible to draw definite conclusions.

Nevertheless, despite these limitations, our results could support the hypothesis that eyedrops combining hyaluronate and natural anti-inflammatory products, such as *Aloe vera*, probably help mitigate the ocular surface inflammation and improve certain aspects of QoL in patients with GTOSD.

As is known, long-term topical therapy for glaucoma, because of the effects of preservatives and active compounds, induces several unfavorable ocular surface changes such as immune activation, epithelial damage, goblet cell loss, and stromal fibrosis, which are largely sustained by several inflammatory mediators, such as interleukins, cytokines, and MMPs [18,19,20]. These alterations not only produce symptoms of OSD and decrease adherence to therapy, but also represent a major risk factor for filtration surgery failure [3,4]. In this perspective, interventions that can safely mitigate the dry eye and ocular surface inflammation, such as lubricants enriched with anti-inflammatory agents, may have different benefits for disease management [3,4].

Both treatments contained PF HMW-HA, which is a well-recognized tear substitute with multiple favorable properties for the ocular surface, including viscoelasticity, water retention, and mucoadhesive effects. HMW-HA has been shown to increase goblet cell density, stimulate MUC5AC secretion, and exert a modulatory effect on ocular surface inflammation [19]. These mechanisms may explain the significant improvements observed in both treatments. However, despite the above-mentioned limitations, *Aloe vera*/0.3% HA showed numerically broader within-group improvements across several parameters, particularly for BCH and CFS, compared with baseline. These changes appeared descriptively larger than those observed in the HA group, although no formal inter-group statistical testing was performed, and were accompanied by a trend toward a reduction in MMP-9 immunoassay positivity. This could be due to the presence of *Aloe vera*, but also to the gel formulation of the eyedrops.

These findings deserve careful attention since previous evidence documented that all IOP-lowering medications induce a significant increase in MMP levels on the ocular surface, which are pro-inflammatory, and a reduction in tissue inhibitors of MMPs (TIMPs) [21,22]. Therefore, treatments that mitigate the MMP/TIMP imbalance could exert favorable effects on the ocular surface [11,12,13,14].

Aloe extracts contain polysaccharides, glycoproteins, and sterols with anti-inflammatory and immunomodulatory effects [23]. In vitro studies showed that *Aloe vera* reduces MMP-9 expression in peripheral blood mononuclear cells, inhibits collagenase and other metalloproteinases through aloins, and decreases pro-inflammatory cytokines (IL-1β, IL-6, TNF-α) and nitric oxide in human corneal cells [14]. A recent randomized trial in patients with dry eye further demonstrated that *Aloe vera*-containing artificial tears decreased tear MMP-9 and IL-6 concentrations and improved tear film stability [11]. Our findings align with the existing literature on *Aloe vera*’s biological properties, extending these findings also to glaucoma patients, a population with high rates of ocular surface damage.

According to Miller et al., a clinically significant improvement in OSDI score was reported in both groups [24]. The clinical improvements observed in tear film stability, corneal staining, conjunctival hyperemia, and symptom scores were evident in both hyaluronate-based formulations, which may contribute to these effects through lubrication, muco-adhesion, and inflammation mitigation. These findings are consistent with previous reports evaluating lubricants and anti-inflammatory adjunctive therapies in OSD, which have similarly documented improvements in tear film stability, corneal staining, and patient-reported symptoms, although with variable effects on inflammatory biomarkers [3,4,5,7,23].

With regard to inflammation, a trend toward reduced tear MMP-9 immunoassay positivity was observed in the *Aloe vera*/0.3% HA group, though this change did not reach statistical significance. This trend should be interpreted cautiously, considering the qualitative nature of the MMP-9 assay, the limited sample size, and the heterogeneity of disease severity among patients enrolled. Moreover, not all patients with GTOSD exhibit elevated tear MMP-9 levels, particularly in milder or clinically stable conditions, which may further limit the sensitivity of this biomarker in detecting treatment-related changes. But on the other hand, we may speculate that, probably, a larger sample size would have better clarified the impact of treatments on MMP-9 levels.

Furthermore, although elevated tear MMP-9 is considered a validated biomarker of ocular surface inflammation, particularly in dry eye disease, recent real-world studies have shown that not all patients with OSD test positive, with positivity rates around 40% and higher sensitivity in severe or more inflamed cases [25,26]. This variability may explain why, in our study, the reduction in MMP-9 immunoassay positivity did not reach statistical significance in either treatment group. Nevertheless, the observed downward trend, especially in the *Aloe vera*/0.3% HA treatment group, suggests a potential anti-inflammatory effect in GTOSD that warrants further investigation in larger, adequately powered studies.

This study has some limitations. First, the sample size and the follow-up period were limited. Therefore, the present results may not be completely representative of the real effects of the use of HMW-HA and *Aloe vera* extracts in patients with GTOSD. Second, patients enrolled were under different IOP-lowering therapy regimens and had different durations of the topical therapy. These aspects, which are normal considering the nature of the study, may hinder the identification of which category of patients would benefit more from treatments. Third, the MMP-9 immunoassay provides only a qualitative assessment of tear MMP-9 levels and may miss subtle quantitative changes. Fourth, we did not perform a prospective, controlled, randomized clinical trial, which would have been the most robust research method to determine whether a cause–effect relationship exists between an intervention and an outcome; moreover, the absence of formal intergroup statistical comparisons limits inference regarding comparative efficacy between the treatment regimens.

However, the strength of an observational, non-interventional registry lies in its structured and prospective design, with standardized inclusion criteria, uniform diagnostic assessments, and longitudinal follow-up, which ensure methodological rigor and allow consistent monitoring of both clinical and inflammatory outcomes in a real-world glaucoma population. Finally, though we found that the use of hyaluronate enriched with *Aloe vera* extracts has potential short-term favorable effects, we cannot provide data on the long-term utility of this treatment in improving the control of the disease, the adherence to therapy, and the surgical outcomes.

In conclusion, this observational sub-analysis of the OSGLA Registry suggests that PF hyaluronate-based lubricants, with or without *Aloe vera* enrichment, are associated with short-term improvements in signs and symptoms of GTOSD. Although the *Aloe vera*/0.3% HA formulation was associated with numerically greater improvements across several outcomes, at this stage, these observations should be interpreted as exploratory rather than definitive. The lack of randomization, absence of inter-group statistical comparisons, short follow-up duration, and reliance on qualitative inflammatory biomarkers warrant caution when interpreting comparative conclusions. Future randomized controlled trials with adequate sample size, longer follow-up, and quantitative biomarker assessment are warranted to clarify the potential added value of *Aloe vera* enrichment and to better define its role in the management of GTOSD.

## Figures and Tables

**Table 1:** Baseline demographic and clinical features.

	GENDER (M/F)	AGE (Years)	IOP (mmHg)	MD (dB)	DURATION OF IOP-LOWERING THERAPY (Years)	N. OF IOP- LOWERING MEDICATIONS	STATUS OF THE LENS (P/Ps)
PF *Aloe vera*/0.3% HA	14/13	73 (77.5–66.5)	18 (20–17)	−3.66 (−2.46/−12.25)	10 (20–5.5)	1 (2–1)	16/11
PF 0.3% HA	8/4	67 (71.5–58.5)	18.5 (20.25–15.75)	−3.16 (−2.09/−4.37)	9 (13.5–5.75)	1 (2–1)	7/5

PF: preservative-free; HA: hyaluronic acid; IOP: intraocular pressure; M/F: male/female; MD: mean deviation; P/Ps: phakic/pseudophakic. Data are expressed as median and interquartile range (Q1–Q3).

**Table 2:** Baseline and one-month follow-up ocular surface clinical tests, questionnaire scores, and MMP-9 immunoassay positivity.

	PF HA 0.3%			PF *Aloe vera*/HA 0.3%		
VARIABLE	Baseline	Follow-Up	*p*-Value	Baseline	Follow-Up	*p*-Value
**BCH** score (0–5)	2.75 (3.20–2.15)	2.25 (2.30–1.90)	*0.014*	2.2 (2.3–1.3)	2.1 (2.2–1.2)	<*0.001*
**CFS** score (0–5)	3.5 (5–2.75)	2.5 (4–2)	*0.014*	3 (4–2)	2 (3.0–1.5)	<*0.001*
**BUT** (s)	6 (6.25–5)	6 (8–5.75)	*0.036*	5 (7–4.5)	7 (8–5.5)	<*0.001*
**STI** (mm)	11.5 (18.25–4.75)	14 (16.25–7.75)	*0.365*	12 (19.5–8)	13.5 (20–10)	*0.034*
**SANDE** score	28.95 (47.6–20.9)	26.86 (36.41–19.90)	*0.009*	36.18 (38.5–20.5)	22.91 (31.5–17.21)	<*0.001*
**OSDI** score	40 (49–19.5)	29 (42–19.75)	*0.005*	29.5 (32.5–19.5)	20 (26.5–18)	<*0.001*
**NEI VFQ-25** score	69.05 (76.25–62.67)	77.22 (78.85–65.40)	*0.064*	61.56 (69.5–38.65)	63 (68.82–39)	*0.22*
**MMP-9 IMMUNOASSAY**	4/8	6/6	*0.479*	11/16	16/11	*0.0736*

PF: preservative-free; HA: hyaluronic acid, BCH: bulbar conjunctiva hyperemia; CFS: corneal fluorescein staining; BUT: break-up time; STI: Schirmer test I; SANDE: Symptoms Assessment iN Dry Eye; OSDI: ocular surface disease index; NEI VFQ-25: National Eye Institute Visual Function Questionnaire; MMP: matrix metalloproteinase. Data are expressed as median and interquartile range (Q1–Q3).

## Data Availability

Data are available on request to the corresponding author.
